# Research on cassava disease classification using the multi-scale fusion model based on EfficientNet and attention mechanism

**DOI:** 10.3389/fpls.2022.1088531

**Published:** 2022-12-22

**Authors:** Mingxin Liu, Haofeng Liang, Mingxin Hou

**Affiliations:** ^1^ School of Electronic and Information, Guangdong Ocean University, Zhanjiang, China; ^2^ School of Mechanical and Power Engineering, Guangdong Ocean University, Zhanjiang, China

**Keywords:** deep learning, classification, EfficientNet, multi-scale feature fusion, attention mechanism

## Abstract

Cassava disease is one of the leading causes to the serious decline of cassava yield. Because it is difficult to identify the characteristics of cassava disease, if not professional cassava growers, it will be prone to misjudgment. In order to strengthen the judgment of cassava diseases, the identification characteristics of cassava diseases such as different color of cassava leaf disease spots, abnormal leaf shape and disease spot area were studied. In this paper, deep convolutional neural network was used to classify cassava leaf diseases, and image classification technology was used to recognize and classify cassava leaf diseases. A lightweight module Multi-scale fusion model (MSFM) based on attention mechanism was proposed to extract disease features of cassava leaves to enhance the classification of disease features. The resulting feature map contained key disease identification information. The study used 22,000 cassava disease leaf images as a data set, including four different cassava leaf disease categories and healthy cassava leaves. The experimental results show that the cassava leaf disease classification model based on multi-scale fusion Convolutional Neural Network (CNN) improves EfficientNet compared with the original model, with the average recognition rate increased by nearly 4% and the average recognition rate up to 88.1%. It provides theoretical support and practical tools for the recognition and early diagnosis of plant disease leaves.

## 1 Introduction

With the current climate posing a threat to human health, vegetation and biodiversity (Bhatti et al., 2022a), and the outbreak of the novel coronavirus pneumonia, major cities across the country have suspended production in order to effectively prevent the spread of the epidemic (Bhatti et al., 2022b). The importance of food is self-evident. In recent years, the planting area has continued to expand, and it is also a key food security crop for smallholder farmers because it can withstand harsh conditions. However, with the increase in cassava planting areas, the disease problem is becoming increasingly prominent. Cassava disease can generally be diagnosed according to the shape, color, and leaf shape characteristics of the disease spots on cassava leaves. Under the influence of environmental factors, cassava disease is more likely to occur, which affects the yield and quality of cassava. According to the different characteristics, cassava disease can be mainly classified into Cassava Bacterial Blight (CBB), Cassava Brown Streak Disease (CBSD), Cassava Green Mottle (CGM), Cassava Mosaic Disease (CMD), etc., which can lead to reduced and/or diseased cassava output. Due to the small number of professional plant personnel of cassava and the lack of professional knowledge of general plant personnel of cassava, the symptoms of cassava leaf disease are not typically studied with good understanding, which can lead to inaccurate and incorrect diagnoses of cassava disease. At the same time, the artificial diagnosis and treatment of cassava leaf diseases not only wastes a lot of manpower and material resources but also results in omission and error from subjective judgment due to the relatively similar characteristics of each leaf disease. The diversity of solutions for cassava disease often prevents effective treatment of cassava, so it is very important to correctly identify the disease. Therefore, as an auxiliary means, computer technology can be applied to help planting personnel determine whether there is cassava disease and of which type, and then the best treatment can be given, to avoid yield decline.

There have been many research achievements in judging plant diseases through traditional machine learning methods. Since they are all based on artificial designs of features, they are inefficient and have a large workload (Pujari et al., 2016). In addition, people tend to rely on experience when selecting features, which is highly subjective and not only consumes manpower but also has a large amount of uncertainty. Combining machine learning with deep learning can solve this problem well. Jamil (Jamil et al., 2021) used artificial neural network (ANN) and support vector machine (SVM) to solve the problem of land classification. When the accuracy of ANN was 82.60% and the accuracy of SVM was 73.66%, they combined the two models and weighted them, and finally the average accuracy reached 86.18%. A CNN can automatically extract image features, greatly reducing the workload, while providing a good research direction for plant disease classification. Meanwhile, K-nearest Neighbor (KNN) classifier (Bazai et al., 2017) and other algorithms are also studied in deep learning in data analysis scenarios, Bhatti (Bhatti et al., 2021a) proposed a method for edge detection of color images by using Clifford algebra and its subalgebra quaternion in image processing, which improved object detection and classification as well as extraction of other features. Bhatti (Bhatti et al., 2021b) also proposed a spatial spectrum HIS classification algorithm – local similarity Projection Gabor Filtering (LSPGF), which uses the reduced-dimension Convolutional Neural Network based on local similarity projection (LSP) and two-dimensional Gabor filtering algorithm. The performance of the proposed method is compared with other algorithms in the public Host Integration Server (HIS) database, and the overall accuracy is better than all datasets. Based on the data information, Bhatti (Aamir et al., 2021; Bhatti et al., 2022c) uses regression analysis algorithm and path analysis algorithm to extract the relationship between variables and get the relationship between algorithms.

With the proposals of AlexNet (Krizhevsky et al., 2012) and Visual Geometry Group Network (VGG) (Simonyan and Zisserman, 2014), the number of network parameters is greatly reduced and the network is more suitable for complex samples under multiple training times, paving the way for deep learning to be applied in future computer vision. In the same year that VGG was proposed, Google proposed GoogleNet (Szegedy et al., 2015). This network adopts the Inception modular structure, by which convolutional kernels of different sizes are used to capture features of feature maps and expand their receptive fields, and then splice the results into channels. Finally, the accuracy of the network is improved by fusing multiple features. In the following years, the proposal of Resnet (He et al., 2016) residual network provided new ideas for the CNN. With the advent of EfficientNet (Tan and Le, 2019), the model has become more capable at capturing features, and its application in computer vision is developing day by day, especially in plant disease recognition.

Hewitt (Hewitt and Mahmoud, 2018) only used a simple shape feature set to judge and recognize relevant plant leaves, in which the feature set included shape features of original leaves and signal features extracted from different convolution models for recognition and obtained good recognition results. In the face of wheat disease leaf identification and differentiation, (Van Hieu and Hien, 2020) used a variety of classification algorithms to compare the prediction accuracy of various neural networks, among which GoogleNet proved to have the highest accuracy of 98%, more suitable for wheat disease detection. As for the detection and recognition of tea diseases, Lee (2018) used Faster Region Convolutional Neural Network (FR-CNN) and candidate objects proposed by Region Proposal Network (RPN) to detect, identify, and distinguish three kinds of tea diseases, with recognition accuracies up to 63%, 81%, and 64%, respectively. Two major crop damage modes in maize production were evaluated, and three commonly used object detectors were evaluated. It was concluded that YOLOv2 had better performance and was more suitable for the assessment of maize growth damage (Turkoglu and Hanbay, 2019). The adversarial robustness of the model (Zoran et al., 2020) was significantly improved by adding an attention mechanism, and the robustness was effectively improved by changing the model expansion steps. On the lightweight model, Wang (Wang et al., 2022) proposed an Individualized activity space modeler (IASM) mechanism to improve the accuracy and efficiency of the model, and achieved the classification accuracy of 92.57% on the self-made data set by using Ghostnet and Weighted Boxes Fusion (WBF) structures. In the classification of banana diseases, Narayanan (Narayanan et al., 2022) combined the mixed algorithm of CNN and Fuzzy Support Vector Machine (FSVM) to classify banana diseases, CNN to detect, and FSVM algorithm to strengthen the classification, and finally achieved good results. In terms of attention mechanism, Zhu (Zhu et al., 2021) added an attentional mechanism module combining Convolutional Block Attention Module (CBAM) and ECA-Net module to the model, which improved the accuracy of the model by 3.4%. Zakzouk, S. (Zakzouk et al., 2021) used AlexNet to classify new rice diseases with an accuracy of 99.71%. The accuracy of the results indicated the feasibility of the automatic rice disease classification system. Tang (Tang et al., 2020) proposed a new two-stage Convolutional Neural Network image classification network. InnerMove, a new image data enhancement method, was used to enhance images and increase the number of training samples, so as to improve the generalization ability of the deep CNN model for image classification tasks. There are also many innovative neural network methods on algorithm models that can provide ideas for classification. At present, the 3D Convolutional Neural Network method (Hameed et al., 2022a) is innovatively used to extract the feature information for the data set. This method can solve the problem with the data better than the pixel-based support vector machine classifier. When it comes to the impact of food security on local and global economies, Mazhar (Hameed et al., 2022b) applied the sequential model in deep learning to classify the outer layer air particles through the analysis and characteristics of objects and fusion. Compared with the existing deep learning method of surface landscape, the accuracy rate reaches 98%.

In view of the above mentioned contents and problems, in order to improve the efficiency of cassava disease classification and recognition, this paper uses the deep convolutional neural network method according to the characteristics of crop disease images in real scenes, takes cassava leaf disease images as the research object, and designs a cassava disease classification model based on multi-scale fusion CNN. A multi-scale fusion module is proposed to extract multi-scale information features of images. Focal loss was adopted to reduce the emphasis on most categories caused by data imbalance, and to solve the problem of low classification accuracy for categories with few samples. CBAM (Woo et al., 2018) module was introduced to obtain key information such as texture and color of cassava leaves, and the result of precise positioning of specific features was achieved.

We enhance and amplify the existing data images and add these images to the existing data set to form new mixed data for training the model. The effectiveness of the proposed model was verified by designing several comparison experiments and comparing them with network models such as Resnet and VGG.

The rest of this article is organized as follows. The Materials and Methods section introduces our proposed cassava disease classification model based on multi-scale fusion Convolutional Neural Network. See the results section for experimental results. Finally, in the conclusion part of the summary of this article.

To summarize, the main contributions of this study are as follows:

(i)a model is developed to recognize cassava disease based on Convolutional Neural Network deep learning.
(ii)the accuracy of cassava disease classification model is evaluated using images taken from nature and artificial enhanced images.
(iii)a lightweight module based on attention mechanism to enhance the classification accuracy of the cassava disease model.

## 2 Method and materials

### 2.1 Cassava disease classification model based on multi-scale fusion Convolutional Neural Network

**Figure 1:**
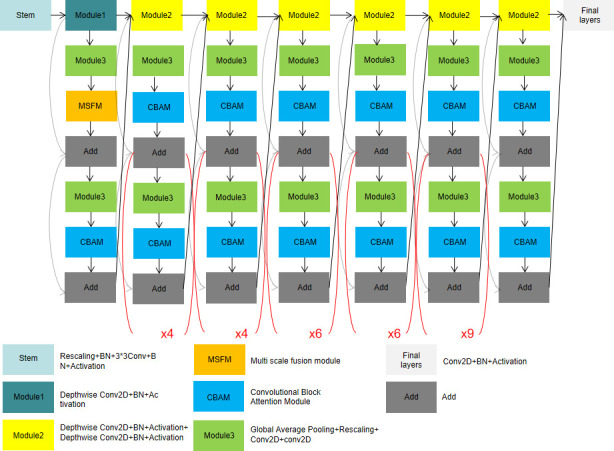
Cassava disease classification model based on multi-scale fusion.

In this paper, a classification model of cassava disease was proposed based on multi-scale fusion (shown in **Figure 1**). The proposed model adopts Efficientnet-B6 as the backbone network, by which a multi-scale fusion module is designed to improve shallow feature extraction. Through CBAM, the channel and spatial weights of subsequent modules are re-calibrated and the classification capacity of cassava leaf disease is enhanced.

#### 2.1.1 EfficientNet

**Figure 2:**
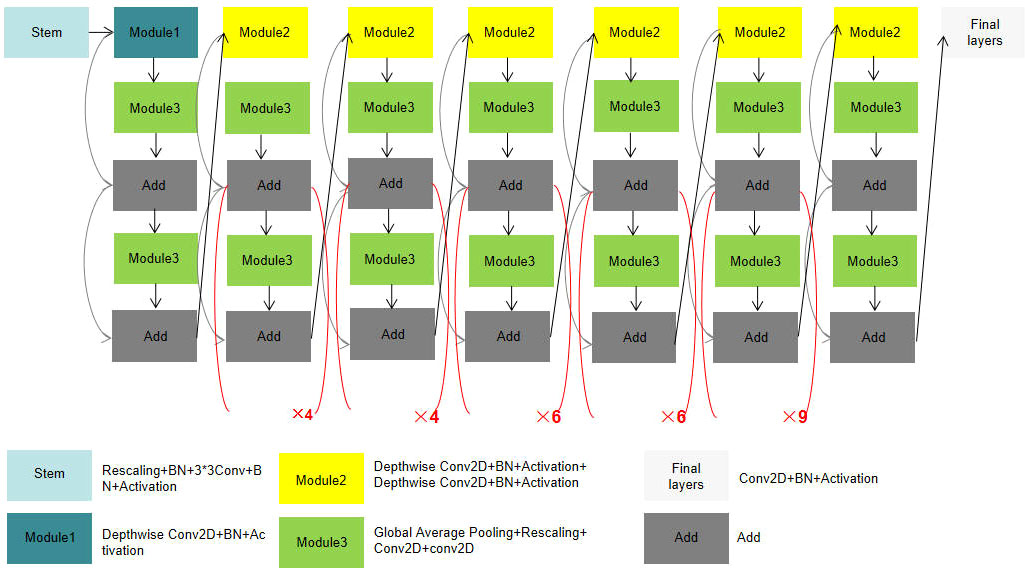
EfficientNet-B6 model structure.

We generally expand the network scale by increasing network depth D, receptive field W, and resolution R. Compared with AlexNet, VGG network convolution kernels were all replaced with smaller 3×3 convolution kernels (including a few 1×1 convolution kernels), which achieved better training results through deeper network structure. However, with the gradual deepening of network depth, network training becomes more difficult due to the emergence of Vanishing Gradient, over-fitting, and other problems. Even if the problem of Vanishing Gradient is solved, the low precision return will lead to high calculation cost and low efficiency of increasing network depth blindly. For another example, MobileNet (Howard et al., 2017) can adjust the number of feature data channels by reducing the amount of computation. However, like deepening the network structure, low precision return will also be found when the width of the network structure reaches a certain level. In the final approach, the neural network can capture finer patterns by using higher-resolution input images. It has developed from 224 × 224 pixels to 229× 229 pixels, or even 512 × 512 pixels. However, accuracy problems are inevitable as the parameter becomes larger. Before EfficientNet, network improvements were generally realized by changing only one of the following variables of network depth, receptive field, and resolution size. However, EfficientNet can obtain better training results by increasing the depth, receptive field, and image resolution through one adjustment. Compared with the aforementioned model, EfficientNet can get a better result because it is capable of adjusting the proportions in three dimensions (shown in **Figure 2**).

#### 2.1.2 Proposed model

##### 2.1.2.1 Multi-scale fusion module

The low-level feature map has a small stride, a large size, and a small receptive field to detect the details of small targets. The high-level feature map has a larger stride, smaller size, larger receptive field, and rich semantic information. The model extracts detailed features such as color and texture from the low-level network and extracts the blade shape feature from the high-level network.

The key features in the map can be selectively enhanced and the features can be accurately located by redistributing both channel and spatial weights through the Attention Mechanism. Compared with the previous EfficientNet model, which did not include the MSFM module, the new model adds modules based on the attention mechanism to allocate computing resources to more important tasks. The operations of different pooling layers in channel and space were added to enhance important features and reduce the proportion of unnecessary features. At the same time, the sensitivity field of the low-level feature map augmented by expansion convolution with different expansion rates can not only extract the details of color and texture, but also fully obtain the context information of the image. In the end, the feature map output obtained by different expansion rates is fused to improve the classification effect.

In the result part, we can see from the comparison experiment that our model has higher accuracy compared with other models, and in the heat map, we can see that it has more accurate judgment on key points.

###### 2.1.2.1.1 Convolutional Block Attention Module 

**Figure 3:**
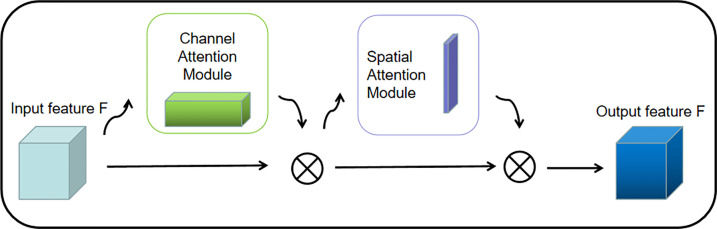
CBAM structure diagram.

The attention mechanism is a relatively efficient data processing method developed in machine learning in recent years, and is widely used in various types of machine learning tasks such as image recognition and natural language processing. When people observe things outside, they usually focus on what they think is important. The attention mechanism focuses on local information that allows the network to achieve better results. Therefore, in this paper, Convolutional Block Attention Module is added before each regularization of the pre-training network and improves the features of the selected maps to increase the accuracy of the model (shown in **Figure 3**).

2.1.2.1.1.1 **Channel Attention Mechanism (CAM)**

**Figure 4:**
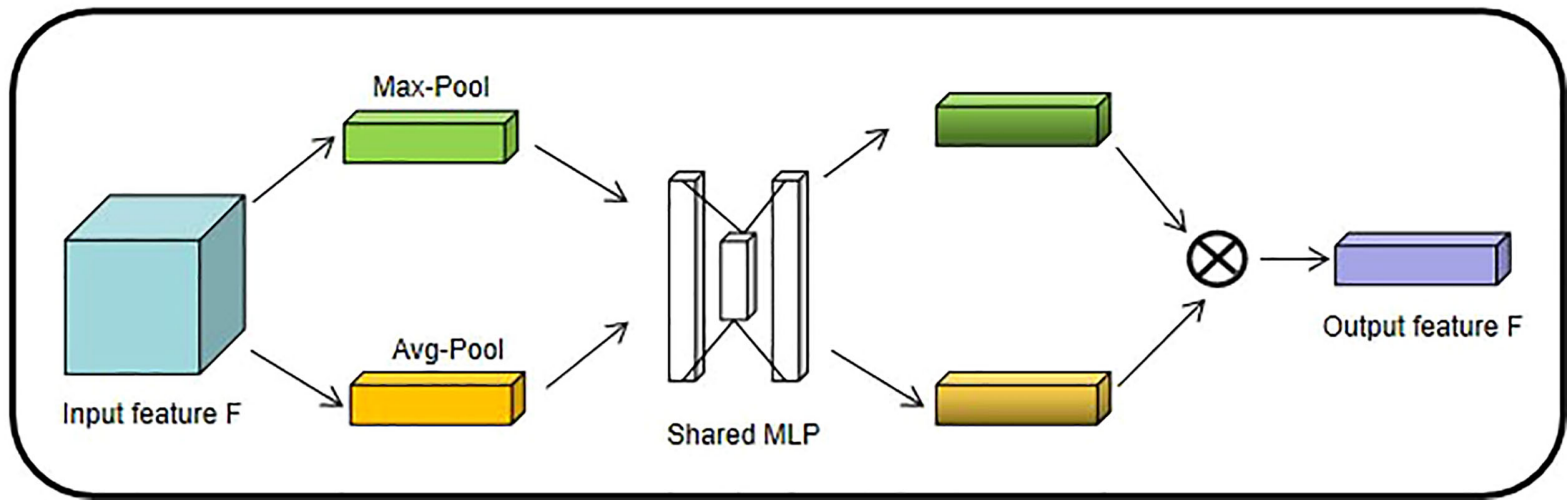
Channel Attention Mechanism structure diagram.

SENet (Hu J. et al., 2018), as the champion network of the 2017 ImageNet classification Contest, is essentially a model based on a channel attention mechanism, which gives rewards and punishments of different weights according to the importance of each feature channel. In this paper, the channel attention mechanism adopts avg-pool and max-pool for fusion. After convolution and activation of the Relu function, the results of the two pooling layers are added together. Finally, it is outputted by the Sigmoid function (shown in **Figure 4**). The size of the input feature map is H×W×C. Firstly, the global maximum pooling layer and average pooling layer are carried out, respectively, to obtain two-channel weight matrices of 1×1×C.

The two matrix results are fed into a two-layer multilayer perceptron (MLP), respectively, and the MLP can share parameters.

After adding the two feature vectors, the weight coefficients are obtained by the Sigmoid activation function again.

The weight coefficient is multiplied by the original input feature to obtain the final output feature.

2.1.2.1.1.2 **Spatial Attention Mechanism (SAM)**

**Figure 5:**
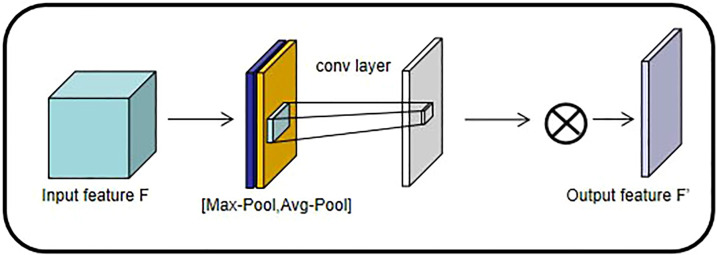
Spatial attention mechanism.

Different from the weight of each feature plane of the channel attention allocation, the spatial attention model is to find the most important part of the network for processing (shown in **Figure 5**).

The input characteristic graph is H×W×C. The Max-Pool and Avg-Pool of one dimension are spliced and dimensionally reduced to generate two one-dimensional feature maps.

The weight parameters are generated by the Sigmoid activation function, and then the final output feature is obtained by multiplying the original input feature.

###### 2.1.2.1.2 Multi-scale fusion module

First, the MSFM redistributes the channel and spatial information through the Convolutional Block Attention Module to enhance the characteristics of small target information. Then Feature Extraction is carried out using dilated convolution of different dilatation rates, and the context information of feature images is fully extracted by expanding different receptive fields. The convolution kernel size of dilated convolution (Yu and Koltun, 2015) is the same as that of ordinary convolution, and the number of parameters in the neural network remains unchanged. The difference lies in that the dilated convolution has a larger receptive field. A 3x3 convolution kernel with an expansion rate of two has the same receptive field as a 5x5 convolution kernel. However, the number of parameters is only nine, much less than the 25 parameters of the 5x5 convolution kernel. The size of the convolution kernel after expansion:


(1) $$k_{d}=\text{k}+\left(\text{k}-1\right)\times \left(\text{r}-1\right)$$

where

*k_d_ = Size of the expanded convolution kernel*

*k = Size of original convolution*

*r = Dilation rate*

The calculation of the receptive field of dilated convolution is as follows:


(2) $${\text{ r}}_{\text{f}}\text{ =[(k+1) }\times \text{(r-1)+k] }\times \text{[(k+1)}\times \text{(r-1)+k]}$$

where

*r_f_ = Receptive field*

*k = Size of original convolution*

*r = Dilation rate*

**Figure 6:**
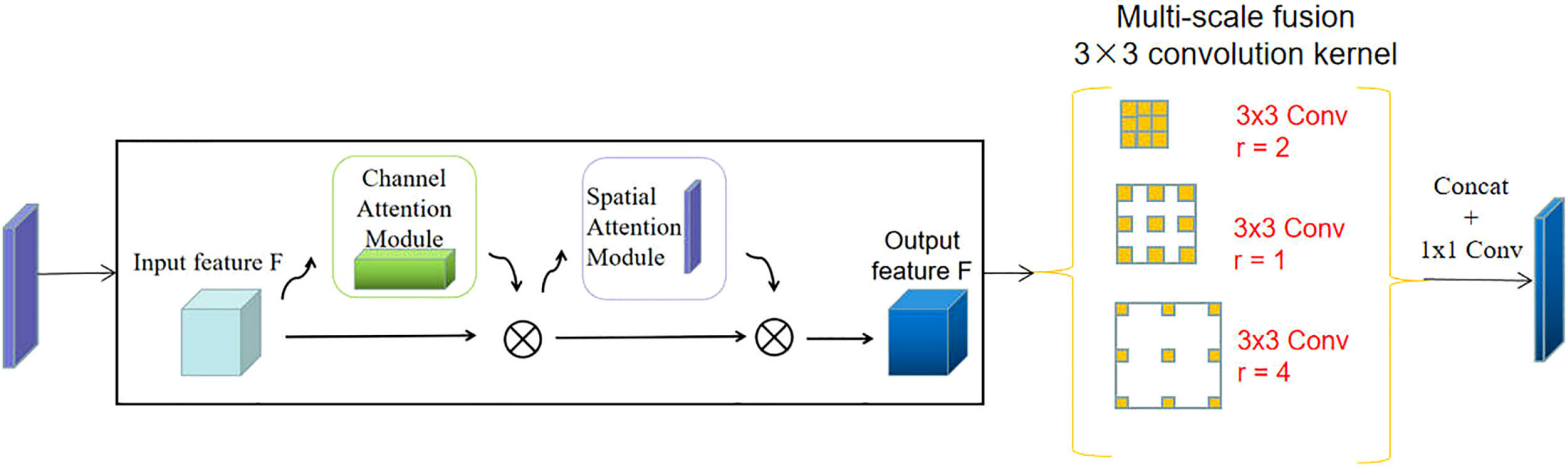
Multi-scale fusion module.

The multi-scale fusion module designed in this paper is shown in **Figure 6**. First, feature maps are learned by the Convolutional Block Attention Module, and weight calibration is carried out for channel and space to strengthen the weight reward of important features of feature maps. The invalid features are punished and the weight is reduced to highlight the important information of the detected image. In this paper, a 3×3 convolution kernel is used for feature extraction, and the expansion convolution with expansion rates of 1, 2, and 4 are used for feature extraction, respectively. The features of different scales are extracted from each layer, and the new features are obtained after dimensionality reduction by fusion.

### 2.2 Loss function

#### 2.2.1 Cross-entropy loss function

In recent years, transfer learning has been widely used in machine learning, which presents satisfactory application results in deep learning. For multi-classification tasks, the cross-entropy loss function (Hu K. et al., 2018) is generally used.

The most commonly used cross-entropy loss function is:


(3) $$CE\left(p_{t}\right)=-a_{t}log\left(p_{t}\right)$$

where

*CE = Loss*

*P_t_ = Predictive value*

*a_t_ = Added parameters that represent weights for different categories*

Cross entropy loss function under multiple classifications:


(4) $$CE=-\sum_{j=1}^{N}a_{t}log\left(p_{t}\right)$$

(b) Focal loss

To a certain extent, traditional methods can solve the problem of fewer categories and unbalanced image distribution, but when there are many easily classified samples, the samples will still dominate the training process, so some difficult-to-classify samples have little chance of gaining the attention of the model. The focus function (Lin et al., 2017) treats the difficult-to-classify samples and the easy-to-classify samples differently, focusing on the difficult-to-classify samples and reducing the weight of the easy-to-classify samples. Therefore, the focus function is adopted as the loss function in this study.

Focal loss adjusts the weights of the difficult-to-classify and easy-to-classify samples in the formula:


(5) $$FL\left(p_{t}\right)=-a_{t}{\left(1-p_{t}\right)}^{\lambda }log\left(p_{t}\right)$$

γ is a constant, and the magnitude of γ determines the weight of small and difficult samples.

When γ<=0, the focusing parameter can be adjusted. When the value of γ is larger, the loss of the sample that is easy to classify is small, and the focus of the model is on the sample that is difficult to classify. This is because when γ is larger, the loss of small samples and difficult samples will be larger, so that they can obtain greater weight.

When γ>1, the training loss of large and simple samples can be reduced, while the loss of small and difficult samples will not be reduced much.

When γ=1, the equation degenerates into the cross-entropy loss function mentioned above.

The focus loss function of multi-classification:


(6) $$FL=-\sum_{j=1}^{N}{\left(1-p_{t}\right)}^{\lambda }log\left(1-p_{t}\right)$$

### 2.3 Input dataset

#### 2.3.1 Data sources and features

**Figure 7:**
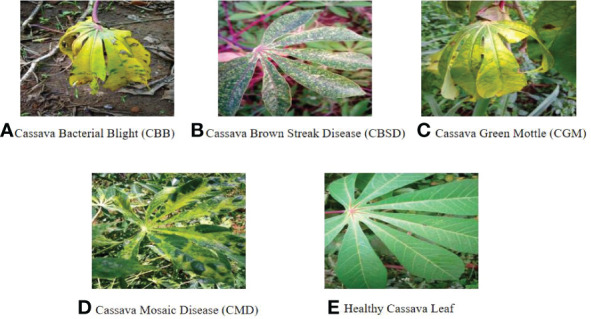
Images of cassava leaf diseases (**A–D** is the disease of four different cassava leaves and e is the healthy leaf).

The data used in this paper are a cassava leaf dataset manually photographed in Uganda and annotated by experts from the National Crop Resources Institute in collaboration with the Artificial Intelligence (AI) Laboratory at Makerere University, Kampala. The data set contains five kinds of cassava leaf images, and disease images are cassava white leaf blight, brown streak disease, green mottling disease, Mosaic disease and healthy cassava leaf images. It can better reflect the characteristics and symptoms of healthy cassava leaves and diseased cassava leaves in natural environment, and it also represents the real and low diagnostic format that farmers need in real life. The dataset includes images taken under field conditions (some of which are shown in **Figure 7**). And images enhanced by data. There are 22,000 of them. Each image has a pixel size of 800×600. The standard input size of neural networks such as ResNet、EfficientNet and Resnet-50 is 224×224 pixels. The whole data set was randomly divided into a training set (90%) and a test set (10%). Therefore, 19,800 images were used for model training, and the remaining 2,200 images were used to test the performance of the model.

#### 2.3.2 Data augmentation

**Figure 8:**
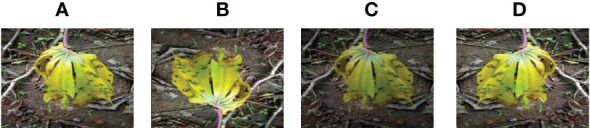
Cassava Bacterial Blight. **(A) **original **(B) **Rotation by 180° **(C)**Brightness reduction **(D) **Horizontal flipping.

**Figure 9:**
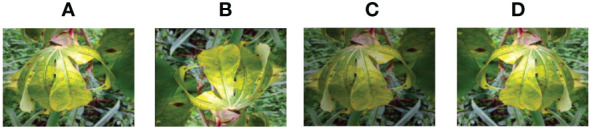
Cassava Brown Streak Disease. **(A) **original **(B) **Rotation by 180° **(C) **Brightness reduction **(D) **Horizontal flipping.

**Figure 10:**
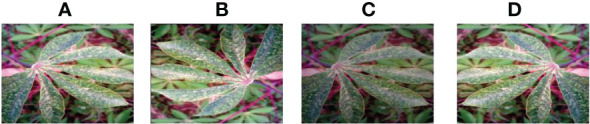
Cassava Green Mottle. **(A)** original **(B) ** Rotation by 180° **(C)** Brightness reduction **(D)**Horizontal flipping.

**Figure 11:**
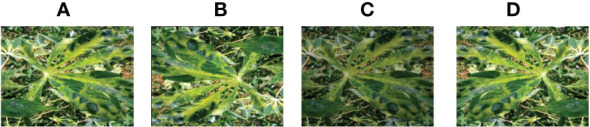
Cassava Mosaic Disease. **(A) **original **(B) **Rotation by 180° **(C)** Brightness reduction **(D) **Horizontal flipping.

**Figure 12:**
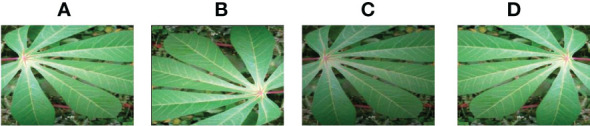
Healthy cassava leaf. **(A) **original **(B) **Rotation by 180° **(C) **Brightness reduction **(D) **Horizontal flipping.

To prevent network overfitting, OpenCV data enhancement was used to expand the data set appropriately. The data set was enhanced by increasing the brightness, decreasing the brightness, and reversing the image, and the data of various cassava leaves amounted to 4000 pieces. The data enhancement methods used are as follows (shown in **Figures 8**–**12**):

Rotation: Rotate the image by 180°.
Brightness reduction: The enhancement factor is 0.7, which means the brightness becomes 70% of the original image.
Horizontal flip: Flip the input image horizontally.

### 2.4 Computer hardware

The proposed method was tested for training and test configuration for neural network models (shown in **Table 1**). This result is achieved in **Table 2**, where PyTorch, as a popular learning framework today, is capable of powerful GPU acceleration and includes deep neural networks. Meanwhile, GPU RTX2080Ti has 11 GB of memory, which can better train the model by adjusting batch size.

**Table 1:** Experimental configuration.

Experimental environment	Model and version
Deep learning framework	Pytorch
Programming language	Python3.7
GPU	NVIDIA GeForce RTX 2080 Ti
The hardware environment	Intel(R) Xeon(R) Silver 4110 CPU @ 2.10 GHz 2.10 GHz

**Table 2:** Classification accuracy of the test set in different models.

Model	Accuracy of the test set/%
VGG-16	70.5
Resnet-18	83.2
Resnet-34	83.9
Resnet-50	85.2
Inception v2	84.1
Inception v3	85.3
MobileNet v3	84.1
ShuffleNet	83.5
EfficientNet-b3	85.5
EfficientNet-b6+	86.5
Our model	88.1

### 2.5 Experimental hyperparameter setting

The cassava leaf data set was divided into a training set, a validation set, and a test set with the ratio of 8:1:1. The training set was trained as an epoch 150 times, 1e-4 was selected as the initial value of the learning rate in the form of a small amount, and the batch size was set to 16. Batch size not only affects the efficiency of the training model but also affects the accuracy. To find a group balance between efficiency and memory capacity, the batch size is used to calculate the batch size.

### 2.6 Model evaluation criteria

Accuracy (%) is used as an evaluation index for multi-classification problems in the laboratory. The accuracy of the experimental model classification can be obtained by removing the number of labels in the test set according to the evaluation criteria. The calculation formula of indicators is as follows:


(7) $$Precision=\frac{TP}{TP+FP}\times 100\%$$

*TP=Positive sample prediction is the number of positive classes.*

*FP=Negative sample prediction is the number of negative classes.*

## 3 Result

### 3.1 Validation and comparison of proposed Convolutional Neural Network

In order to verify that the improved model in this paper has better image recognition ability compared with the traditional model, this paper uses multiple groups of comparative experiments. The experimental results are shown in **Table 2**. According to the evaluation index results, as shown in the figure above, the improved model is compared with the model with better performance recently. Among them, the accuracy rate of the model proposed in this paper is 88.1% in the test set separated from the data set, both of which are better than the previous models. To verify that the improved model in this paper has better image recognition ability compared with the traditional model, multi-group comparative experiments were carried out (shown in **Table 2**).

### 3.2 Ablation experiments

**Figure 13:**
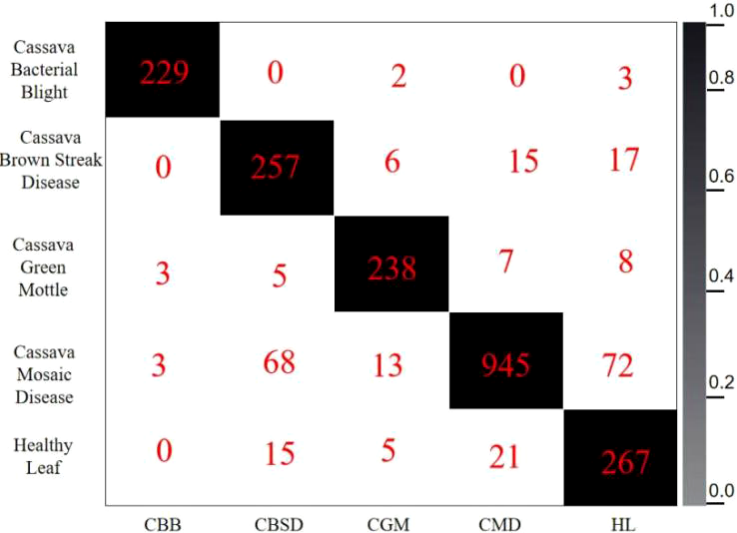
Confusion matrix for the test set.

The confusion matrix (Song et al., 2015) is also one of the evaluation indicators of the classification model (shown in **Figure 13**). The confusion matrix parameters are converted by classification report. The parameters are given as follows:

(i)The percentage of the total that all predictions are correct, as in Equation 1:


(8) $$Accuracy=\frac{TP+TN}{TP+TN+FP+FN}$$

(ii)The proportion of those correctly predicted to be positive that are actually positive, as in Equation 2:


(9) $$Recall=\frac{TP}{TP+FN}$$

(i)The proportion of correct positive precisions to total positive precisions, as in Equation 3:


(10) $$Precision=\frac{TP}{TP+FP}$$

The column labels of the confusion matrix represent the predicted cassava leaf disease type, and the sum of the corresponding row values represents the sum of the samples of this type. The diagonal line indicates the number of correctly predicted labels. Each value on the diagonal line indicates the number of correctly predicted labels. The value at the intersection of the columns represents the value of the corresponding tag predicted. If it is not on the diagonal, it can be seen as the number of wrongly predicted tags. The darker diagonal suggests the better model. In the classification results, the judgment accuracy is high, and most of the results of the test set are concentrated on the diagonal of the confusion matrix. The identification accuracy of all kinds of blades is greater than 90%. However, the identification accuracy of Cassava Mosaic Disease in the test set is lower than that of other diseases, and it is easy to misjudge it as other cassava leaf diseases. By observing the confusion matrix, it can be found that the pictures of Cassava Mosaic Disease can be easily identified as Cassava Bacterial Bligh and Cassava Green Mottle, because the symptoms of these three diseases are relatively similar, so classification errors are prone to occur. The obfuscation matrix of the improved model presented in this paper has better performance and a higher average recognition rate.

### 3.3 Visual output comparative analysis

**Figure 14:**
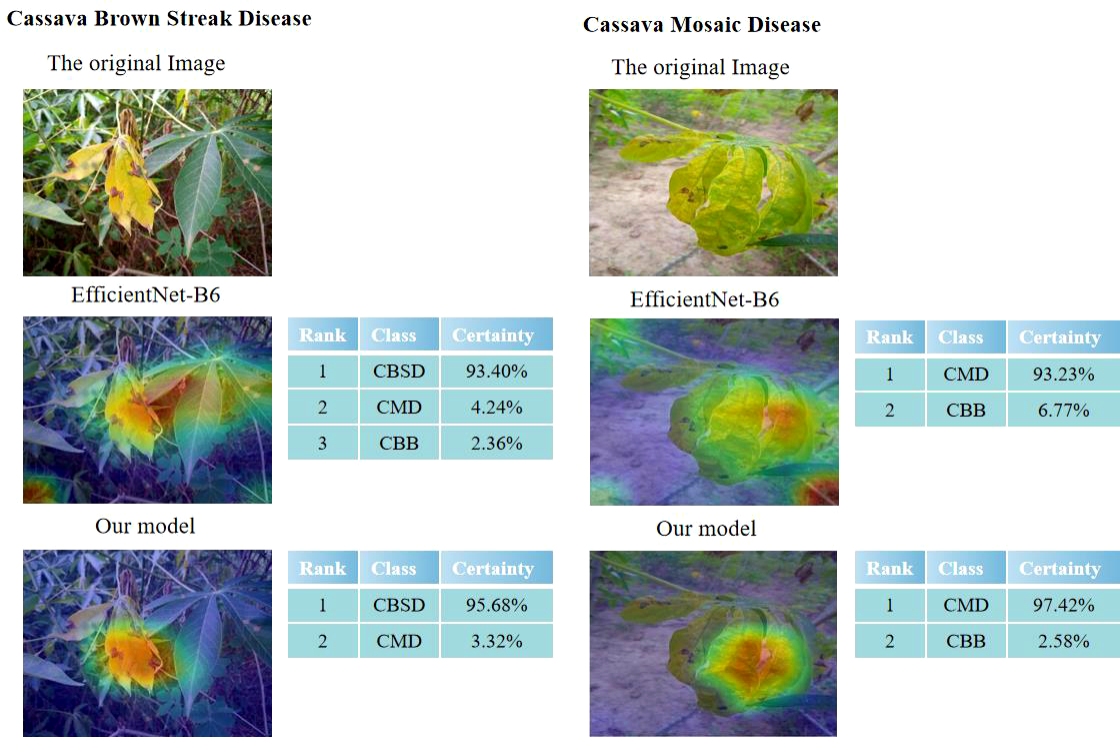
Visual comparison of EfficientNet-B6 and our model.

Class Activation Mapping (CAM) was used to visualize each trained model (Zhou et al., 2016) to better compare the expression process of image features between an improved network and a traditional network. Feng (Feng et al., 2022) used the Grad-CAM thermal map of interpretative analysis, the feature extraction effect of the model can be better expressed. In visualization, the thermal map and the original image are superimposed (shown in **Figure 14**). This is a visual output of the original image and Efficientnet-B6 and our model, respectively. The darker the color is, the larger the value and the more feasible it will be to serve as the judgment. Compared with the model in this paper, our model has a stronger feature extraction ability and better effects in the face of Cassava leaf disease, which not only extracts different colors of cassava leaf disease but also better captures features in the context. In addition, it can achieve more accurate extraction of key information. It can also be seen from the comparative experiment that the original model has some judgment errors in the color discrimination of leaves and the discrimination ability of background information, while the improved one has a better grasp of sample information and a better capture effect for judgment features.

## 4 Conclusions

Cassava, as one of the three varieties of potato, is an important food. Compared with the diagnosis of cassava disease by human, the identification of cassava disease by computer and deep learning method not only has low cost and higher accuracy than manual diagnosis, but also greatly reduces the efficiency. The multi-scale cassava leaf classification model proposed in this paper can better ensure the safety and efficiency of cassava food production and judge the cassava disease type more accurately. Compared with the diagnosis of cassava leaf disease using the human eye, the identification of cassava leaf disease by computer and deep learning method is characterized by lower cost, higher accuracy, and greatly increased efficiency. In this paper, a multi-scale fusion module was proposed, and the Focal Loss function and CBAM module were introduced. An optimization network model of a multi-scale fusion network based on EfficientNet and attention mechanism was proposed. The model was used to train the cassava leaf disease data set and compare with the EfficientNet, ResNet50, and VGG16 networks. The experimental results show that the improved network proposed in this paper has higher precision and better generalization ability. The problem of uneven data was solved by changing the loss function, the distinguishing ability of cassava leaf diseases was improved through an attention mechanism, and the recognition ability of the model was enhanced by multi-layer fusion. According to the pricing standard of the model, the model proposed in this paper can be used for image recognition of Cassava leaf disease.

Since our model adds the lightweight module MSFM based on EfficientNet, our model can be installed on mobile devices, such as microprocessors. Due to the large-scale application of 5th Generation Mobile Communication Technology (5G) (Johannes et al., 2017), there is efficient transmission. According to the improvement of the hardware configuration of mobile terminal equipment, the image to be detected can be uploaded to the cloud server for processing, and then the recognition and classification results can be returned to the terminal. For some cassava planting technicians, when they have doubts about cassava disease judgment, the mobile terminal deployed with the model can detect and classify cassava disease in real time, which is equivalent to having a valuable consulting tool. In the future, a cassava disease detection system can be developed based on the classification results of cassava diseases, which can judge the disease categories and provide corresponding management methods. This can greatly improve the planting efficiency of cassava, improve the production efficiency of cassava, achieve scientific and technological progress of agriculture, and promote agriculture into the era of intelligence. Although the model in this study achieved a good success rate in a limited number of cassava diseases, cassava diseases are not limited to these diseases. To improve this, more images can be collected in different cassava planting areas and field conditions, and the model can be more effective in identifying cassava diseases based on field conditions by amplifying the dataset.

## Data availability statement

Publicly available datasets were analyzed in this study. This data can be found here: https://www.kaggle.com/competitions/cassava-leaf-disease-classification/data.

## Author contributions

ML: Modeling and experimental design of cassava leaf disease; HL: Experimental design and data analysis and arrangement; MH: AI model selection and debugging. All authors contributed to the article and approved the submitted version.
